# Hysteresis and Stochastic Fluorescence by Aggregated Ensembles of Graphene Quantum Dots

**DOI:** 10.1021/acs.jpcc.2c02472

**Published:** 2022-06-16

**Authors:** Nikita Belko, Lena Golubewa, Vyacheslav Chizhevsky, Sopfy Karuseichyk, Dmitry Filimonenko, Marija Jankunec, Hamza Rehman, Tatsiana Kulahava, Polina Kuzhir, Dmitri Mogilevtsev

**Affiliations:** † B. I. Stepanov Institute of Physics, NAS of Belarus, Nezavisimosti ave. 68, 220072 Minsk, Belarus; ‡ A. N. Sevchenko Institute of Applied Physical Problems, Belarusian State University, Kurchatova str. 7, 220045, Minsk, Belarus; § Department of Molecular Compounds Physics, State Research Institute Center for Physical Sciences and Technology, Vilnius, 10257, Lithuania; ∥ Université Paris-Saclay, CNRS, ENS Paris-Saclay, CentraleSupélec, LuMIn, 91190, Gif-sur-Yvette, France; ⊥ Institute of Biochemistry, Life Sciences Center, Vilnius University, Vilnius, 10257, Lithuania; # Institute of Photonics, Department of Physics and Mathematics, University of Eastern Finland, Joensuu, 80101, Finland; ○ Laboratory of Nanoelectromagnetics, Institute for Nuclear Problems of Belarusian State University, Minsk, 220006, Belarus

## Abstract

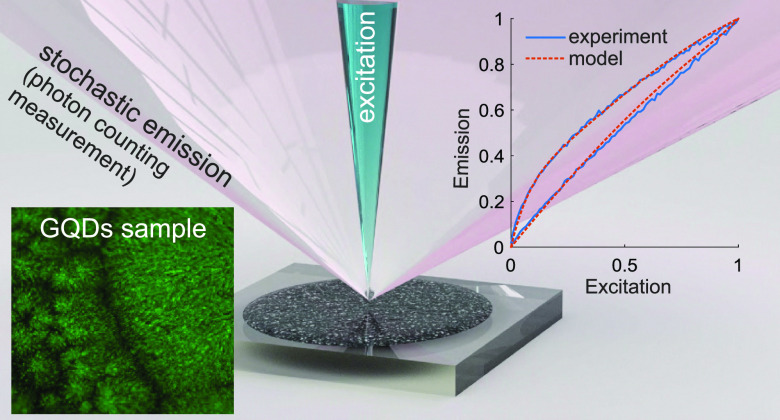

“Blinking” behavior of fluorophores, being harmful for the majority of super-resolved techniques, turns into a key property for stochastic optical fluctuation imaging and its modifications, allowing one to look at the fluorophores already used in conventional microscopy, such as graphene quantum dots, from a completely new perspective. Here we discuss fluorescence of aggregated ensembles of graphene quantum dots structured at submicron scale. We study temperature dependence and stochastic character of emission. We show that considered quantum dots ensembles demonstrate rather complicated temperature-dependent intermittent emission, that is, “blinking” with a tendency to shorten “blinking” times with the increase of temperature. We verify “blinking” mechanism demonstrating hysteresis of the optical response under pulsed excitation timed to expected rates of dots transition to “dark” nonemitting states. Experimental results are well fitted by a simple qualitative model of transitions to the “dark” states. The obtained results suggest that this type of standardized quantum dots and even their submicron-size agglomerations can be useful as controlled fluorophores for super-resolution microscopy and, particularly, for SOFI-like microscopy.

## 1 Introduction

Starting from theoretical proposals in the 90s, super-resolution fluorescent microscopy had rapidly become both a flourishing research field and indispensable, highly useful practical diagnostic tool suitable for both lateral and in-depth imaging and *in vitro* and *in vivo* diagnostics.^1−3^ The basic instruments of all the fluorescent microscopy varieties are nanoscale particles, fluorophores. In addition, among potential fluorophores for super-resolution microscopy, graphene quantum dots (GQDs) have quite a distinct place. GQDs can be of small and well-controllable size (say, 1.5–2.5 nm),^4−7^ exhibit tunable fluorescence,^4,5^ are water-soluble,^4,8^ possess high photostability,^5−7,9−11^ and have low toxicity.^4,7,10−14^ These features make GQDs a biocompatible fluorophore suitable for biosensing and bioimaging.^6−11,13−17^ The well established scalable synthesis methods allow large-scale fabrication of comprehensively characterized GQDs [e.g., used in the present study green luminescent, water-dispersed, CAS 7440–44–0, Sigma-Aldrich, U.S.A.^18^] that make them standardized nanoparticles for biomedical applications.^19−23^ In addition, GQDs can cross the blood–brain barrier,^13,14^ implying neuroimaging applications. Naturally, GQDs were already used as fluorophores for super-resolution microscopy.^9,24^

A typical feature of quantum dots is stochastic fluorescence intermittency, that is, so-called “blinking”. A quantum dot might randomly “switch off” and remain unresponsive for a time, much exceeding typical emission time and then “switch on” again. Whereas this effect might be harmful for imaging, there are super-resolution methods explicitly exploiting this feature for enhancing the resolution. First of all, one must mention here so-called stochastic optical fluctuation imaging, or SOFI,^25^ and its modifications.^26^ Notice that quantum dots also allow enhancing the resolution by exploiting the nonclassical (namely, single photon) character of the emitted field.^27^

Basically, SOFI works by measuring correlation functions of different orders and calculating cumulants. In the simplest SOFI case, one just uses the fact that the cumulant of the sum of independent sources equals the sum of cumulants from each source.^25^ To be able to infer more information about the emitters configuration with increasing order of the measured correlation functions, “blinking” statistics should be non-Gaussian.^28^ Single GQDs can exhibit “blinking”^9,29^ and are potentially useful for super-resolution imaging based on the stochastic fluorescence intermittency.

Fluorescent properties of GQDs strongly depend on the preparation method.^4,5,7,13,15^ In this work, we study fluorescent properties of GQDs agglomerates, naturally formed, for example, by drying drops of aqueous suspension of GQDs on the silicon substrate. We show that such GQDs ensembles do demonstrate features usually associated with single GQDs, in particular, stochastic intermittency. This ensemble intermittency is complicated occurring on several time-scales. So, being structured on a small enough scale, agglomerations of GQDs can still be used for super-resolution imaging, in particular for SOFI-like methods. We also discuss a rather pronounced temperature dependence of the intermittency (in particular, considerable shortening of “dark” times with moderate temperature changes). To verify the blinking mechanism of transition to the “dark” states, we have studied the GQDs fluorescence excited by triangular pulses with duration corresponding to the typical time-scales of transitions to the “dark” states. We have demonstrated expected hysteresis behavior of the GQDs fluorescence corresponding to the simple qualitative model of transition to the “dark” states.^30,31^

The outline of the paper is as follows. In section 2 we describe our GQDs samples and experimental techniques used to characterize them. In section 3.1 we report results on spatial and spectral properties of the used GQDs samples, and section 3.2 is devoted to the time-dynamics of emission and its temperature dependence. In section 3.3 we discuss the mechanism and manifestation of stochastic intermittency (i.e., “blinking”), and in section 3.4 we describe probing the mechanism underlying stochastic intermittency by triangular excitation, revealing hysteresis in dependence of fluorescence on the pumping field intensity. We also propose a simple qualitative model of transitions to the “dark” states and back and show that the results are well fitted by it.

## 2 Materials and Methods

To prepare high-concentrated samples of GQDs for optical measurements, 50 μL of a 1 mg·mL^–1^ aqueous suspension of GQDs (Sigma-Aldrich) was drop-cast onto a clean silicon substrate and allowed to dry in the dark. A low-concentrated GQDs sample for atomic force microscopy (AFM) was prepared by spin-coating 15 μL of a 50 μg·mL^–1^ suspension at 1500 rpm for 40 s onto a clean silicon substrate. Sample preparation and measurements were carried out at room temperature (20 ± 1 °C).

Fluorescence imaging of the GQDs sample was performed using an inverted Eclipse Ti–U (Nikon) microscope. CFI Plan Fluor DLL (Nikon) objectives with a 40× magnification and 0.75 numerical aperture or 10× magnification and 0.3 numerical aperture were used. The GQDs sample was covered with a cover glass. The excitation light source was a LED with a peak wavelength of 470 nm. The fluorescence signal was detected with a DU-897E-CS0-UVB (Andor) EMCCD. Fluorescence emission and excitation spectra of the GQDs on a silicon substrate were recorded using a Fluorolog 3 (Horiba Scientific) spectrofluorimeter.

Scanning electron microscopy (SEM) was carried out using an S-4800 (Hitachi) electron microscope at an operating voltage of 15 kV in the secondary electrons detection mode. AFM imaging was performed in the tapping mode on a Dimension Icon (Bruker) scanning probe microscope system using FESPA-V2 (Bruker) silicon AFM probes. The scans were performed at a 512 pixel or higher resolution with a scan rate of 0.3–0.5 Hz.

**Figure 1:**
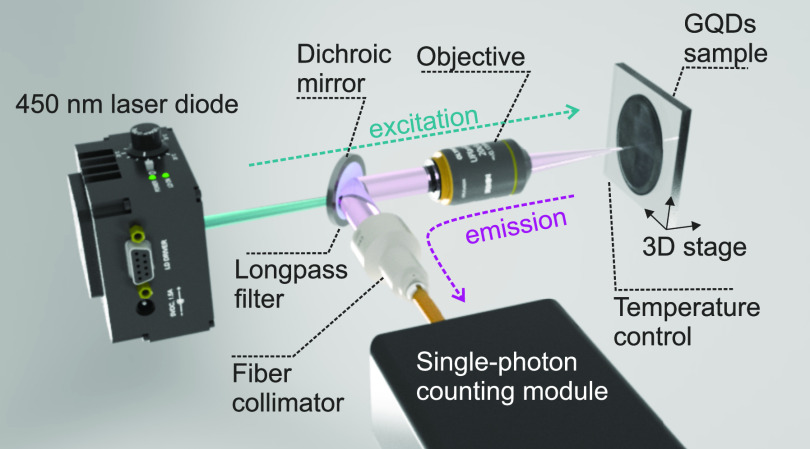
Schematic of the optical setup used to measure emission signal from the GQDs. The GQDs sample on a silicon substrate was temperature controlled and mounted on a 3D stage. The sample was irradiated with a 450 nm diode laser through the objective. The emission from the GQDs was collected by the same objective, separated from the scattered excitation light by the dichroic mirror, and additionally cleaned up by the long-pass filter. The emission signal was collected by the fiber collimator and guided into the single-photon counting module via the multimode fiber.

Emission dynamics for the GQDs was investigated using a custom-made optical setup (Figure 1, notice that it is quite simple to build and cost-effective compared to those required for studying emission of individual GQDs). Fluorescence was excited by a 450 nm laser diode (Thorlabs, PL450B). The temperature of the diode was set to 25 °C and controlled by a thermoelectric temperature controller (Thorlabs, TC200). The laser diode was operated in the CW or pulsed mode. In the pulsed mode, the current of the laser diode was driven by symmetrical triangular pulses. The duration of the laser pulses was 80 s if not noted otherwise. The laser beam irradiated the sample at normal incidence through a plan fluorite microscope objective (Olympus) with a 20× magnification and 0.5 numerical aperture resulting in a 0.2 mm spot on the sample. The power density varied from 7 to 110 W·cm^–2^.

Fluorescence emission of the GQDs was collected using the same objective. The reflected laser light was separated from the fluorescence signal using a dichroic mirror (Thorlabs, DMLP505). The fluorescence signal from the sample was reflected by the dichroic mirror into a fiber collimator and directed into a multimode optical fiber. The optical fiber was connected to an ID100-MMF50 (Quantique) single-photon counting module with a dark count rate of 32 Hz. The fluorescence signal was additionally cleaned up by a long-pass edge filter (Semrock, 488 nm EdgeBasic) placed between the objective and the fiber collimator. The counts from the single-photon counting module were recorded using an HS5-540 (TiePie) oscilloscope. The pulses from the oscilloscope were then converted to delta pulses, and the mean frequency of the pulses was determined by averaging over a 10 ms time frame. The mean frequency of the counts was directly proportional to the fluorescence intensity since the detector was not saturated under the given experimental conditions. A schematic of the optical setup is shown in Figure 1.

The GQDs sample was positioned in space with 0.5 μm precision using a stepper-motor driven stage. *X* travel allowed us to place the sample in the focal plane of the objective. *Y* and *Z* travel were used to collect fluorescence of the GQDs from different areas of the sample. The temperature of the sample was varied using a thermoelectric element and measured using a thermistor.

## 3 Results and Discussion

### 3.1 Spatial Distribution and Steady-State Spectral Properties of Samples

**Figure 2:**
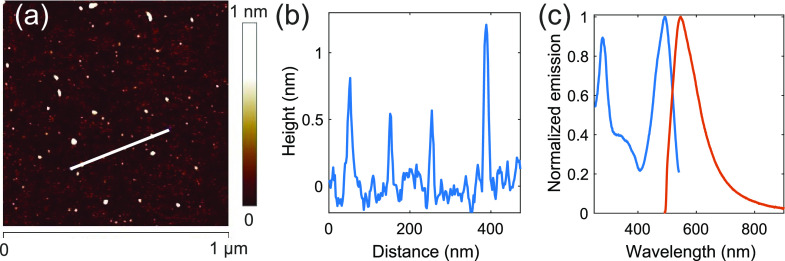
(a) AFM micrograph for the low-concentrated GQDs sample prepared by spin-coating the diluted GQDs suspension onto a silicone substrate. (b) The height profile along the white line shown in panel (a). (c) Typical normalized fluorescence emission spectrum excited at 450 nm (right orange curve) and normalized fluorescence excitation spectrum monitored at 570 nm (left blue curve) measured for a high-concentrated GQDs sample.

First, we have taken steps to characterize individual GQDs used in our work. The dimensions of individual GQDs were determined using AFM. For that purpose, the low-concentrated GQDs sample was prepared by spin-coating. The AFM micrograph for the sample (Figure 2a,b) revealed particles with a diameter of 17.0 ± 5.6 nm and a height of 0.67 ± 0.43 nm (averaged over 87 particles). The impact of the AFM tip convolution effect was not taken into account.

For characterization of fluorescence and SEM measurements, a number of similar high-concentrated GQDs samples were prepared by drop-casting.

The fluorescence emission spectra of prepared GQDs samples excited at 450 nm exhibited a single asymmetric band (FWHM 3350 cm^–1^) peaked at 546 nm (Figure 2c, red curve). Bands at 279 and 494 nm as well as a broad feature in the 300–400 nm range were present in the fluorescence excitation spectrum monitored at 570 nm (Figure 2c, blue curve). The presence of multiple bands in the excitation spectrum indicates the involvement of several electronic transitions in the GQDs emission. In the following experiments, the fluorescence of the GQDs was excited at 450 nm, that is, via the lowest-energy electronic transition (fluorescence excitation band in 400–550 nm range) to minimize heat production.

**Figure 3:**
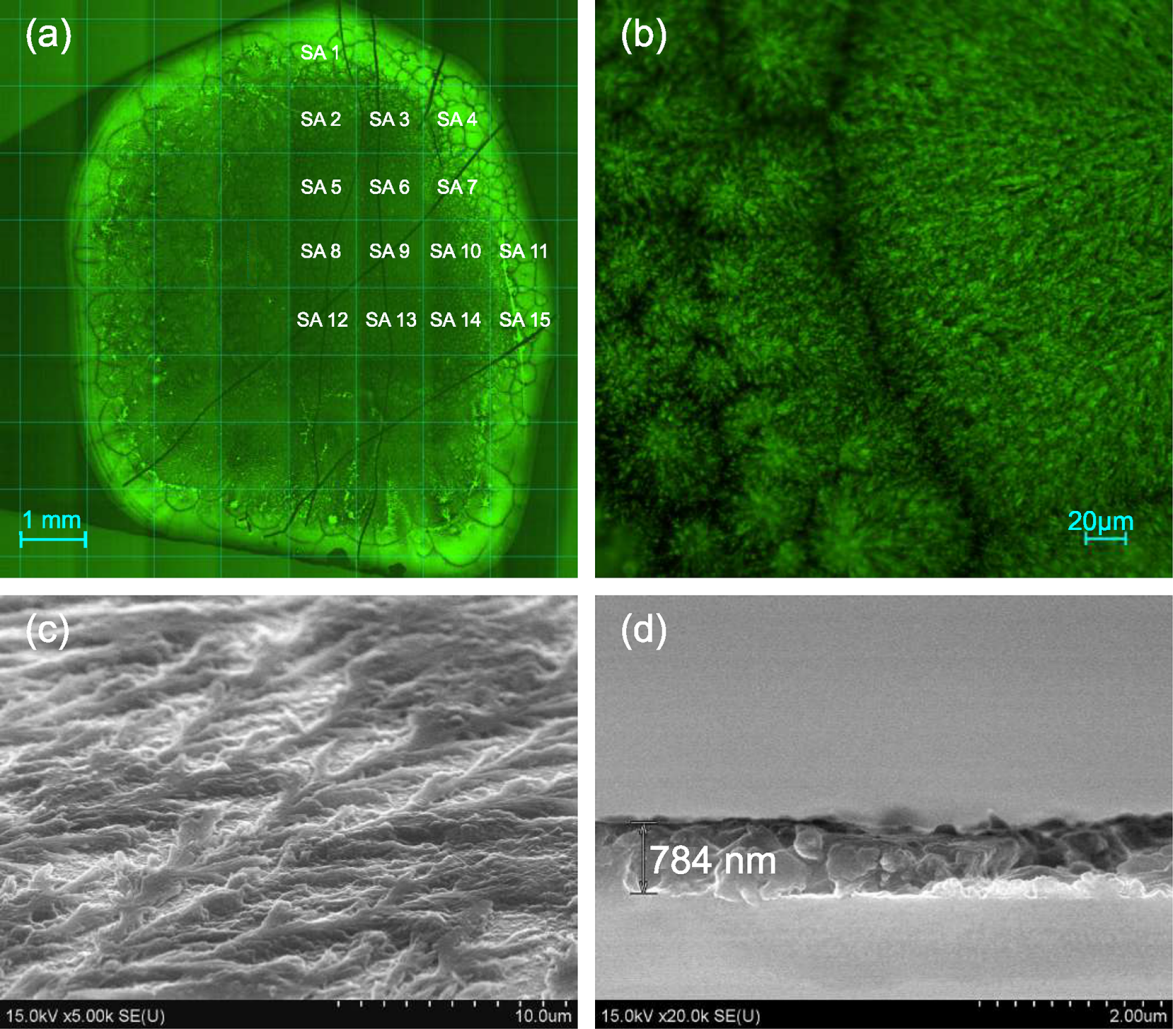
(a) Fluorescence microscopy image for a high-concentrated GQDs sample. The numbers indicate different sample areas (SAs) used to measure fluorescence. (b) Fluorescence microscopy image for an edge part of the GQDs sample shown at higher magnification. (c) SEM micrograph showing surface morphology for the central region of a typical sample (such as SA numbered 8 in panel (a)). (d) SEM cross section for the typical GQDs sample.

The fluorescence microscopy images (Figure 3a,b) for the samples revealed green fluorescent ∼100 μm agglomerations structured on the submicron scale. As shown in our previous work,^32^ the as-received GQDs suspension might contain a water-soluble polymer, which improves the stability of GQDs in water, but does not affect the optical properties of GQDs in the visible range. As a result, the effect of the polymer in suspensions can be ignored. However, when the GQDs suspension is dried on the substrate, the presence of the polymer plays a critical role along with the action of surface tension forces. It influences the GQDs spatial organization and distribution on the surface. The polymer also supports the formation of ensembles of GQDs, which is not observed in aqueous suspensions. These ensembles mimic, for example, the formation of agglomerates in living cells as in the intracellular space nanoparticles are often surrounded by a lipid bilayer of the transport vesicle. Investigation of the behavior of these ensembles opens the way to understanding the optical properties of groups of particles. The methods of super-resolution microscopy can thus be improved by taking into account the behavior of particle ensembles surrounded by a complex organic environment (components of living cells).^33−37^

GQDs distribution on the silicon surface is complex. The agglomerations visible in fluorescence images in Figure 3a,b along the edge of the drop are due to the action of the surface tension forces during the drying. The observed tree- and bush-like structures both along the drop edge and inside the droplet can be attributed to the GQDs ensembles embedded in the polymer. Nevertheless, the concentration of the GQDs is considerably higher at the edge of the sample. The top-view SEM micrograph (Figure 3c) confirms that the GQDs are covered with the polymer that helps the particles to group into extended, highly branched structures and provides GQDs distribution inside the droplet. Using the side-view SEM images, we also determined that the GQDs-enriched polymer film was ∼0.8 μm thick (Figure 3d). Comparison of the fluorescence microscopy images and SEM micrographs allows concluding that on-surface distribution of GQDs is secured by the polymer, which is responsible for GQDs ensembles formation and spatial organization.

### 3.2 Emission and Its Temperature Dependence for CW Excitation

For investigating optical emission, the sample was scratched to provide for the reference points, and different sample areas (SAs) were chosen for performing measurements (see Figure 3a). Different SAs are referred to using the numbers shown in Figure 3a.

Time-dependent fluorescence emission of different areas of the GQDs sample was first studied under CW irradiation. Here, we compare data obtained for SA 5, 6, and 7 characterized by different concentrations of the GQDs (Figure 3a). Data for other SAs can be found in the Supporting Information.

**Figure 4:**
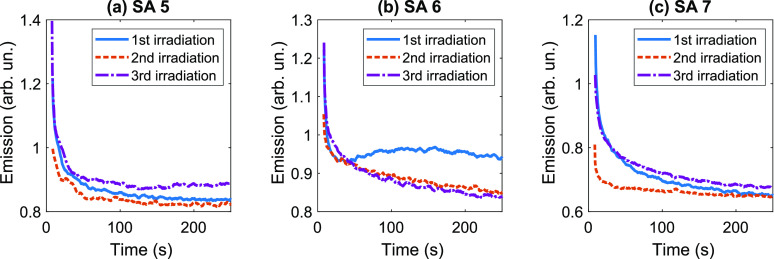
Emission time traces for SAs 5 (a), 6 (b), and 7 (c) measured under three consecutive CW irradiations. Delay between the irradiations was 45 min. The first irradiation (solid blue curves) was performed after keeping the sample in the dark for 2 days. The sample was cooled to 15 °C and heated back to room temperature between the second (dashed orange curves) and third (dash-dotted purple curves) irradiations. The excitation power density was 110 W·cm^–2^.

Emission time traces were recorded during three consecutive 250-s long CW irradiations. The excitation power density was 110 W·cm^–2^. Delay between the irradiations was 45 min. The first irradiation was performed after keeping the sample in the dark for 2 days. Between the second and third irradiations, the sample was cooled to 15 °C and heated back to room temperature. The emission time traces are shown in Figure 4 (data for other SAs are shown in Figure S1). The concentration of the GQDs had a substantial influence on the shape of the emission time traces. For all SAs, the emission signal exhibited rapid decay during the initial 50 s of irradiation. The time trace for SA 6 demonstrated growing emission signal in a certain time frame.

The overall emission signal recorded during the second irradiation was lower for most SAs. Remarkably, cooling the sample to 15 °C and heating it back to room temperature allowed us to recover the fluorescence of the GQDs. For some SAs, the emission signal recorded after the cooling even exceeded the signal obtained during the first irradiation.

**Figure 5:**
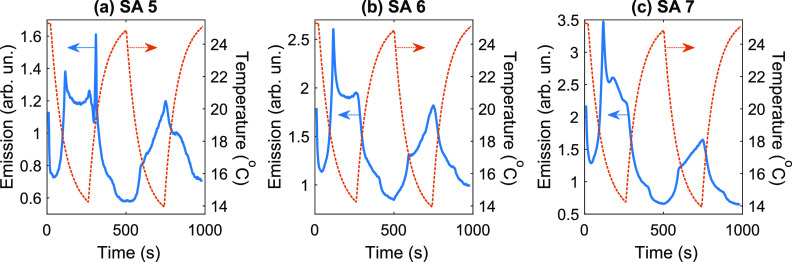
Emission time traces measured under CW irradiation and changing temperature for SAs 5 (a), 6 (b), and 7 (c). Cycle of cooling for 4 min and subsequent heating for 4 min was performed twice. The solid blue curves show the emission time traces. The dashed orange curves display the temperature of the sample vs time. The excitation power density was 110 W·cm^–2^.

Then we attempted to control the emission behavior of the GQDs by varying the temperature of the sample. All studied SAs exhibited a remarkable increase in the fluorescence intensity when the temperature dropped from 25 to 14 °C (Figures 5 and S2). The location of the SAs influenced the shape of the emission time traces.

**Figure 6:**
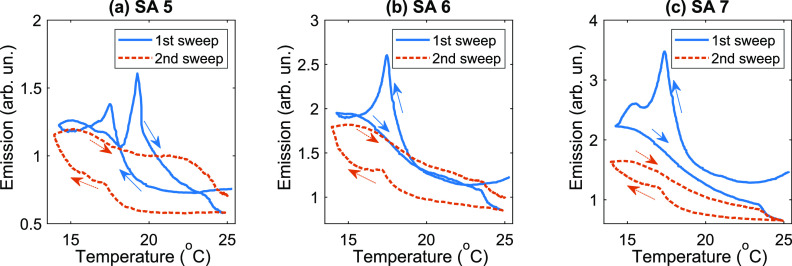
Emission intensity as a function of the temperature for SAs 5 (a), 6 (b), and 7 (c). The solid blue (dashed orange) curves show data for the first (second) cycle of cooling for 4 min and subsequent heating for 4 min. The data in Figure 5 and in this figure are identical but shown in different axes. The excitation power density was 110 W·cm^–2^.

These data were also analyzed in axes displaying the emission signal as a function of the temperature (Figures 6 and S3). It can be seen that the emission signal exhibited dynamic hysteresis as the temperature varied (which is expected for graphene structures, see, for example, ref (38).). The changes in fluorescence intensity were delayed relative to the changes in temperature.

### 3.3 Stochastic Intermittency

The fluorescence emission of the GQDs demonstrated a number of interesting features: the decay of the fluorescence intensity under CW irradiation; dependence of the fluorescence time traces on the SA; the complex response to changes in temperature. The observed behavior could be explained in terms of transitions of the GQDs between emissive (“bright”) and nonemissive (“dark”) states under irradiation. An increase (a decrease) in the fluorescence signal on dropping (rising) temperature seems to indicate that transitions to the “dark” states are inhibited (enhanced) at lower (higher) temperatures.

Stochastic fluorescence intermittency was previously reported for single GQDs^9,29^ and their close relative, carbon nanodots (CNDs).^6,17,39,40^ Oxygen functional groups (especially hydroxyl and carbonyl) are often attached to the surface of GQDs^8,12,41−44^ and CNDs^6,45^ and were suggested to be the origin of their fluorescence.^6,29,42,43,46^ The surface groups are believed to act as emissive or nonradiative trap states.^29,41,47,48^ Electron transfer and charge redistribution on the surface of CNDs seem to be responsible for their transitions between “bright” and “dark” states.^6,17,48^ To the best of our knowledge, the literature has not discussed the mechanism of stochastic fluorescence intermittency in GQDs.

As shown in our previous work, a variety of oxygen functional groups (carbonyl, hydroxyl, epoxy, etc.) are present on the surface of the GQDs under study.^32^ Fluorescent properties of these GQDs are changed after hypochlorite-induced oxidation. These findings indicate that the oxygenated functional groups might be the origin of the fluorescence intermittency in the GQDs.

The observed response of the fluorescence of the GQDs to changing temperature is consistent with the assumption that electron transfer plays a major role in transitions of the GQDs between “bright” and “dark” states. As the thermal energy of electrons is reduced, the electron transfer processes are inhibited, and transitions to the “dark” states become less probable. Complex fluorescence time traces measured on changing temperature (Figures 5 and 6) might indicate that there are several types of surface trap states responsible for the transitions of the GQDs to the “dark” states. Some of the trap states might be deactivated at reduced temperatures. Thermally activated trap states in GQDs were reported before.^42^

### 3.4 Hysteresis under Pulsed Excitation

The presented results do not allow us to clarify the processes underlying the stochastic fluorescence intermittency in the GQDs. Nevertheless, one can build a quite intuitive picture of “blinking” arising from GQDs transition to “dark” states, and simple fluorescence dynamics associated with such transition. The validity of this intuitive picture can be experimentally accessed in a quite straightforward manner. Let us take a pulsed excitation with a slowly rising front of the pulse and a slowly decreasing tail. Let us assume that the sample is “bleached” due to the excitation, that is, GQDs are going into “dark” states, and the percentage of the “bleached” GQDs is proportional to the excitation intensity. Then, if the pulse length is adjusted to the typical transition time to the “dark” state, one should expect hysteresis of the fluorescence in dependence on the excitation intensity. A similar method was applied to probe dark-state dynamics in organic molecules.^49^

Curiously, despite this simple intuitively expected manifestation of hysteresis, to the best of our knowledge, there exist no reports on observing dynamic hysteresis of photoluminescence for GQDs. The closest work that we could find on hysteresis in optical properties of graphene structures is dynamic hysteresis of transmittance for graphene oxide suspensions.^38^

#### 3.4.1 Experimental Results

First, let us discuss the observed fluorescence hysteresis phenomena. Emission time traces were measured for three consecutive sweeps of the excitation power. The delay between the sweeps was 10 min. Before the first sweep, the sample was kept in the dark for 2 days. Between the second and third sweeps, the sample was cooled to 15 °C and heated back to room temperature. The excitation pulse duration was 80 s. The peak excitation power density was 110 W·cm^–2^.

**Figure 7:**
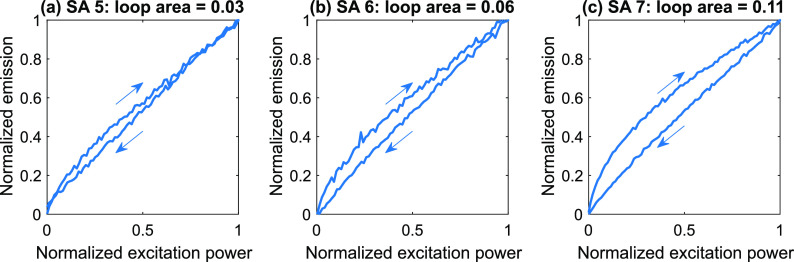
Hysteresis loops measured on sweeping excitation power density for SAs 5 (a), 6 (b), and 7 (c). The excitation pulse duration was 80 s. The peak excitation power density was 110 W·cm^–2^.

The first sweep of the excitation power resulted in pronounced hysteresis behavior of the emission signal (Figures 7 and S4). To compare hysteresis for different SAs, the areas of normalized hysteresis loops were calculated (shown in Figures 7 and S4). Higher concentration of the GQDs at the edge of the sample favored stronger hysteresis.

**Figure 8:**
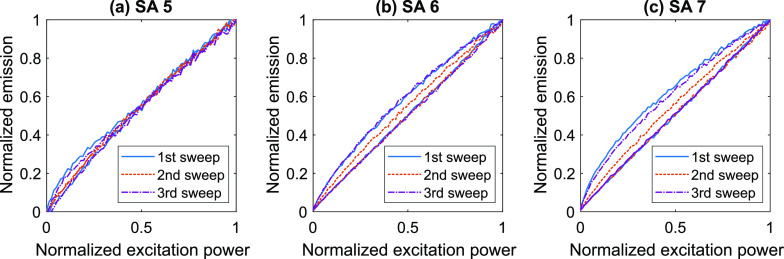
Hysteresis loops for SAs 5 (a), 6 (b), and 7 (c) measured during three consecutive sweeps of the excitation power density. Delay between the measurements was 10 min. The first measurement (solid blue curves) was performed after keeping the sample in the dark for 2 days. The sample was cooled to 15 °C and heated back to room temperature between the second (dashed orange curves) and third (dash-dotted purple curves) measurements. The excitation pulse duration was 80 s. The peak excitation power density was 110 W·cm^–2^.

During the second sweep, the areas of the hysteresis loops dropped drastically compared to the first sweep (Figure 8). It should be noted that complete recovery of the hysteresis loops was observed at least 12 h after sweeping the excitation power. We attempted to obtain a faster recovery by cooling the sample to 15 °C and heating it back to room temperature. Cooling the sample indeed allowed us to achieve a much faster recovery (Figure 8).

**Figure 9:**
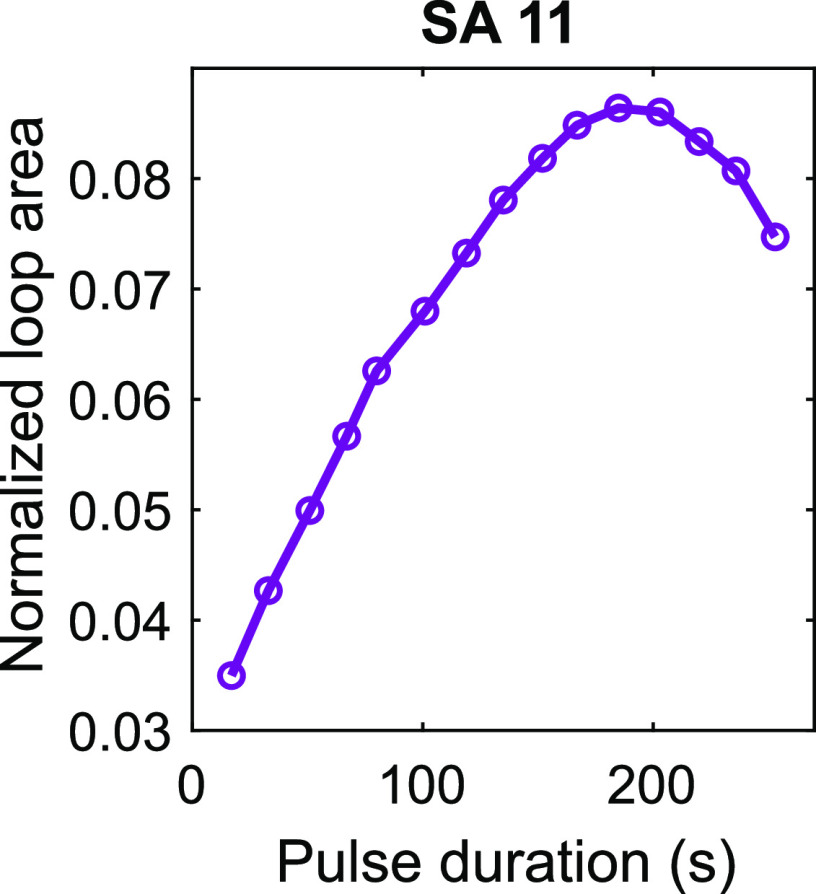
Area of normalized hysteresis loop vs the duration of excitation pulses measured for SA 11. The peak excitation power density was 7 W·cm^–2^).

After cooling the sample, the hysteresis was recovered only partially (Figure 8). To ensure a faster and fuller recovery of the emission signal between sweeps, the excitation power density was reduced by a factor of 15 (down to 7 W·cm^–2^). After three cycles of changing the temperature and sweeping the excitation power density, the loop areas stabilized and displayed virtually identical values for the following cycles. This feature permitted us to measure the dependence of the hysteresis loop area on the duration of the excitation pulses and, thus, to demonstrate how the excitation pulse length could be fitted to the “dark” state transition time. The sample was cooled to 15 °C and heated back to room temperature between consecutive sweeps. Unfortunately, we were unable to obtain satisfactory data for SA 5, 6, or 7. The dependence of the hysteresis loop area on the duration of the excitation pulses for SA 11 is shown in Figure 9. It can be seen that the loop area was growing on increasing pulse duration, reached its peak value for the pulse duration of 175 s, and then started to fall.

The observed behavior of the hysteresis loop area versus pulse duration is the reflection of the “dark”-state dynamics in the GQDs. For short excitation pulses, only a small fraction of the emitters entered the “dark” states. Lengthening the excitation pulses resulted in a larger hysteresis loop area due to increased population of the “dark” states. For even longer excitation pulses, a dynamic equilibrium between population and depopulation of the “dark” states was probably established yielding less pronounced hysteresis.

The hysteresis in the emission of the GQDs could be due to a local change in the temperature of the sample induced by the absorbed excitation light.

#### 3.4.2 Model

Now let us demonstrate that experimentally observed fluorescence intensity behavior can be quite well captured by the following simple model:
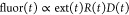
1where ext(*t*) is the time dependence of the excitation field power density. In the experiment we have chosen triangular pulses of the length 2*t*_max_, that is, ext(*t*) ∝ *t* for *t* ∈ [0, *t*_max_], and *I*_pump_(*t*) ∝ *t*_max_ – *t* for *t* ∈ [*t*_max_, 2*t*_max_]. The function *D*(*t*) describes transitions of GQDs to “dark” states; the function *R*(*t*) describes the process of GQDs returning to the “bright” state.

**Figure 10:**
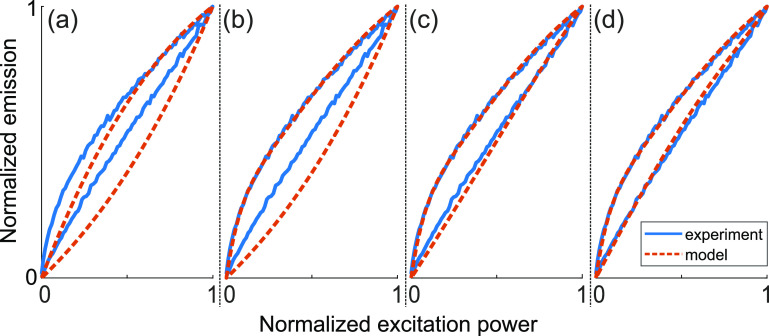
(a) Monoexponential model of eqs 1 and 2, with *R*(*t*) = 1 and *a*_1_ = 0; (b) Biexponential model of eqs 1 and 2, with *R*(*t*) = 1 and *a*_1_ = 1, *a*_2_ = 0.1; (c) Biexponential linear return model of eqs 1 and 2, with *a*_1_ = 1, *a*_2_ = 0.1, and *R*(*t*) given by eq 3 with *b* = 0.6 and *x* = 1; (d) Biexponential nonlinear return model of eqs 1 and 2, with *a*_1_ = 1, *a*_2_ = 0.1, and *R*(*t*) given by eq 3, with *b* = 0.6 and *x* = 1.2.

In Figure 10 one can see the results of fitting the experimental hysteresis curve obtained for the region SA 7. The process of the GQDs transition into “dark” states was described by the following expression
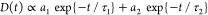
2where the rates τ_1,2_ describe the rates of transitions to “shallow dark” and “deep dark” states. These rates were estimated from the experimental data for SA 7. A sum of two exponentials was fitted to the emission time trace shown in Figure 4c, which yielded τ_1_ = 5 s and τ_2_ = 77 s (*R*^2^ = 0.997). The coefficients *a*_1,2_ represent respective proportions of GQDs going to “shallow dark” and “deep dark” states.

Figure 10a shows that just a single-exponential approximation describing transition to the “deep dark” state with the transition time τ_2_ already gives a rather decent description of the hysteresis process. Assuming presence of both “shallow dark” and “deep dark” states, one quite precisely fits the dynamics corresponding to the first half of the triangular excitation pulse (Figure 10b).

An even more precise fit can be obtained if one accounts for the possible return of GQDs from “dark” states when the excitation intensity decreases. It can be captured with the following diffusive-type time dependence of the function *R*(*t*):
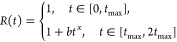
3where we have taken *b* = 0.6 [s]^−*x*^. Quite a good agreement for the returning branch of the hysteresis curve is given already by a simple linear dependence, that is, *x* = 1 (Figure 10c). Assuming some nonlinearity, *x* = 1.2, the fit becomes almost precise (see Figure 10d). One might surmise that diffusive-type return of GQDs to “bright” state is connected with the effects of heating/cooling of the sample as the result of the photoexcitation. Of course, the influence of a local change in the temperature might affect also the hysteresis in general, say, the rates of going to “dark” states and returning from them.

## 4 Conclusions

In this work we have studied fluorescence emission of a large ensemble of the GQDs under CW and pulsed excitation using a simple optical setup. The fluorescence signal was found to be decreasing over time under CW irradiation and recovering in the dark. As the temperature of the GQDs sample dropped, the fluorescence intensity increased exhibiting different patterns depending on the sample area. The mechanism underlying stochastic intermittency of the fluorescence signal was uncovered by demonstrating hysteresis behavior of the fluorescence power in dependence of the excitation power density by pulsed triangular excitation. The response of the fluorescence intensity to irradiation seems to be occurring due to transitions of the GQDs between the “bright” and the “dark” states. The population of the “dark” states can be controlled by changing temperature.

The obtained results suggest that this type of GQDs and even nanosize agglomerations of them can be useful as controlled fluorophores for super-resolution microscopy, and particularly for SOFI-like microscopy. Future work will entail investigating stochastic fluorescence intermittency of the GQDs at the single-particle level and using the GQDs for super-resolution imaging of biological samples.

## Supporting Information Available

Additional emission time traces under CW irradiation and constant or changing temperature as well as hysteresis loops under excitation by triangular pulses (PDF)
