# Supplementary material for: Hysteresis and Stochastic Fluorescence by Aggregated Ensembles of Graphene Quantum Dots
Source: J Phys Chem C Nanomater Interfaces. 2022 Jun 16;126(25):10469–77. doi: 10.1021/acs.jpcc.2c02472 (PMC9251769; doi:10.1021/acs.jpcc.2c02472)
Supplement: Supplementary file 1 — jp2c02472_si_001.pdf [file jp2c02472_si_001.pdf]

Supporting Information for "Hysteresis and Stochastic Fluorescence by Aggregated Ensembles of Graphene Quantum Dots"

Nikita Belko,[†,‡] Lena Golubewa,[¶] Vyacheslav Chizhevsky,[†] Sopfy Karuseichyk,[§] Dmitry Filimonenko,[†] Marija Jankunec,[‖] Hamza Rehman,[⊥] Tatsiana Kulahava,[#] Polina Kuzhir,[⊥,@] and Dmitri Mogilevtsev[*,†,@]

†*B. I. Stepanov Institute of Physics, NAS of Belarus, Nezavisimosti ave. 68, 220072 Minsk, Belarus*

‡*A. N. Sevchenko Institute of Applied Physical Problems, Belarusian State University, Kurchatova str. 7, 220045 Minsk Belarus*

¶*Department of Molecular Compounds Physics, State Research Institute Center for Physical Sciences and Technology, Vilnius, 10257, Lithuania*

§*Université Paris-Saclay, CNRS, ENS Paris-Saclay, CentraleSupélec, LuMIn, 91190 Gif-sur-Yvette, France*

‖*Life Sciences Center, Department of Bioelectrochemistry and Biospectroscopy, Vilnius University, Vilnius, 10257, Lithuania*

⊥*Institute of Photonics, Department of Physics and Mathematics, University of Eastern Finland, Joensuu, 80101, Finland*

#*Laboratory of Nanoelectromagnetics, Institute for Nuclear Problems of Belarusian State University, Minsk, 220006, Belarus*

@*These authors contributed equally*

E-mail: d.mogilevtsev@ifanbel.bas-net.by

Phone: +37517 2708025

Here we present additional data on the GQDs emission. Figure S1 demonstrates emission time traces for the SAs 1–4, 8–9, and 15. It can be seen that the shape of the emission time traces strongly depends on the SA and, consequently, on the concentration of the GQDs.

Figure S2 shows the emission time traces under CW irradiation and changing temperature. Figure S3 depicts identical data but in different axes (emission intensity vs. temperature). Again, the response of the emission signal to changing temperature differs drastically for different SAs.

Figure S4 demonstrates hysteresis in emission excited by triangular pulses. The normalized areas of the hysteresis loops are substantially greater for the SAs located close to the edge of the sample (SAs 1, 3, 4, 10, 11, 14, and 15).

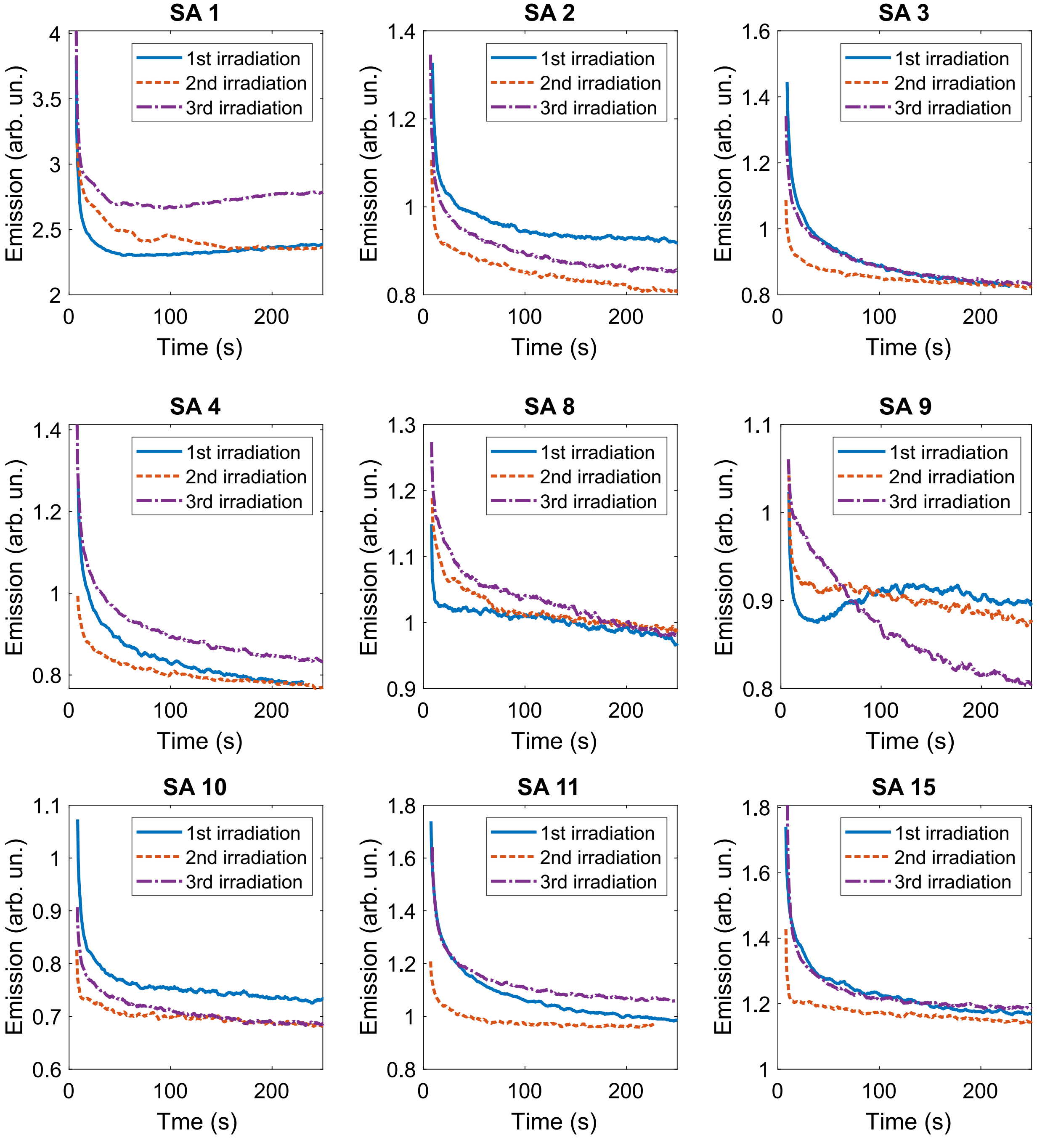

Figure S1: Emission time traces for SAs 1–4, 8–11, and 15 measured under three consecutive CW irradiations. Delay between the irradiations was 45 min. The first irradiation (blue curves) was performed after keeping the sample in the dark for 2 days. The sample was cooled to 15°C and heated back to room temperature between the second (orange curves) and third (purple curves) irradiations.

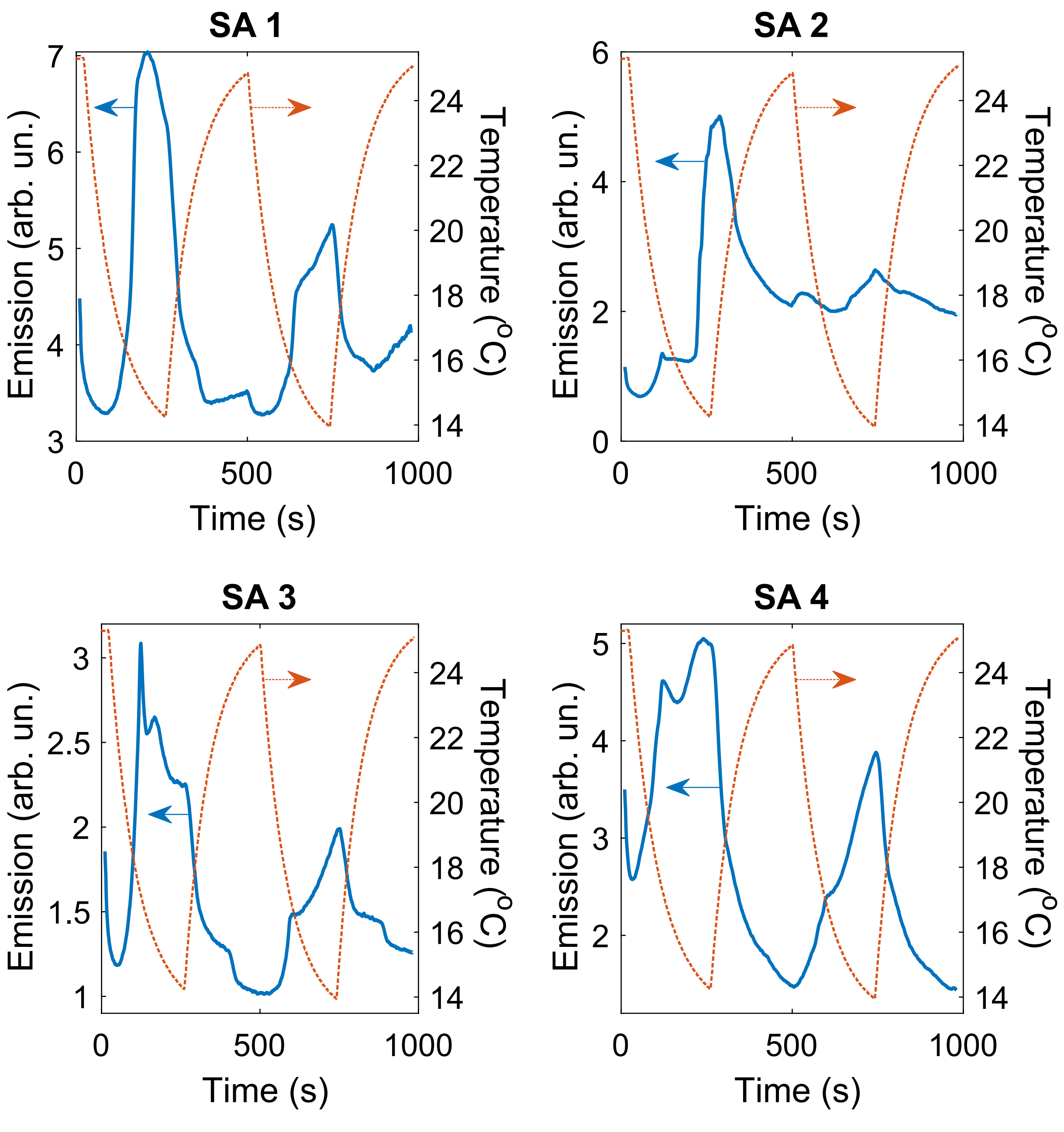

Figure S2: Emission time traces measured under CW irradiation and changing temperature for SAs 1–4. Cycle of cooling for 4 min and subsequent heating for 4 min was performed twice. The blue curves show the emission time traces. The orange curves display the temperature of the sample vs. time.

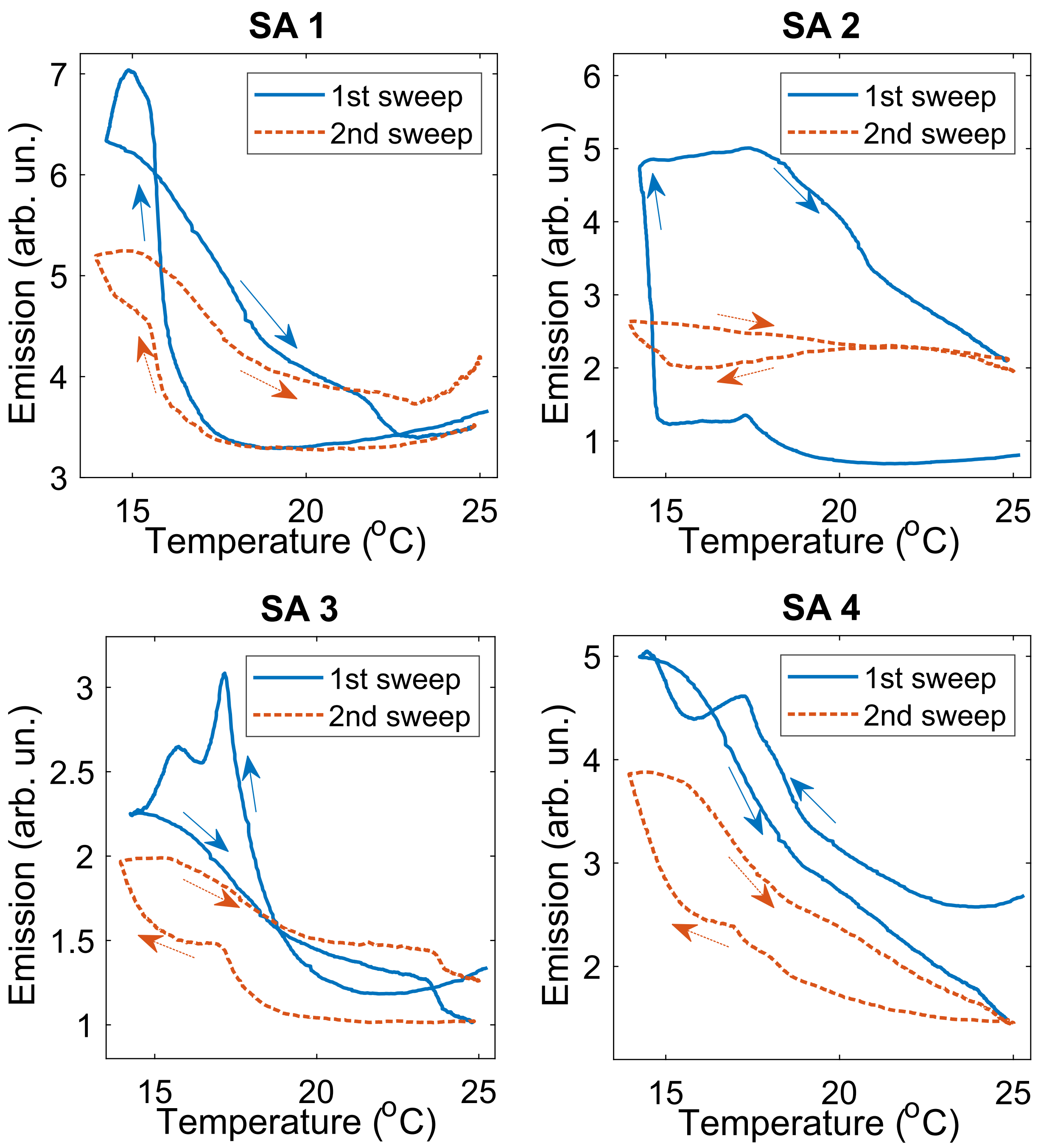

Figure S3: Emission intensity as a function of the temperature for SAs 1–4 (SAs are indicated on the legends). The blue (orange) curves show data for the first (second) cycle of cooling for 4 min and subsequent heating for 4 min. The data in Figures S2 and S3 are identical but shown in different axes.

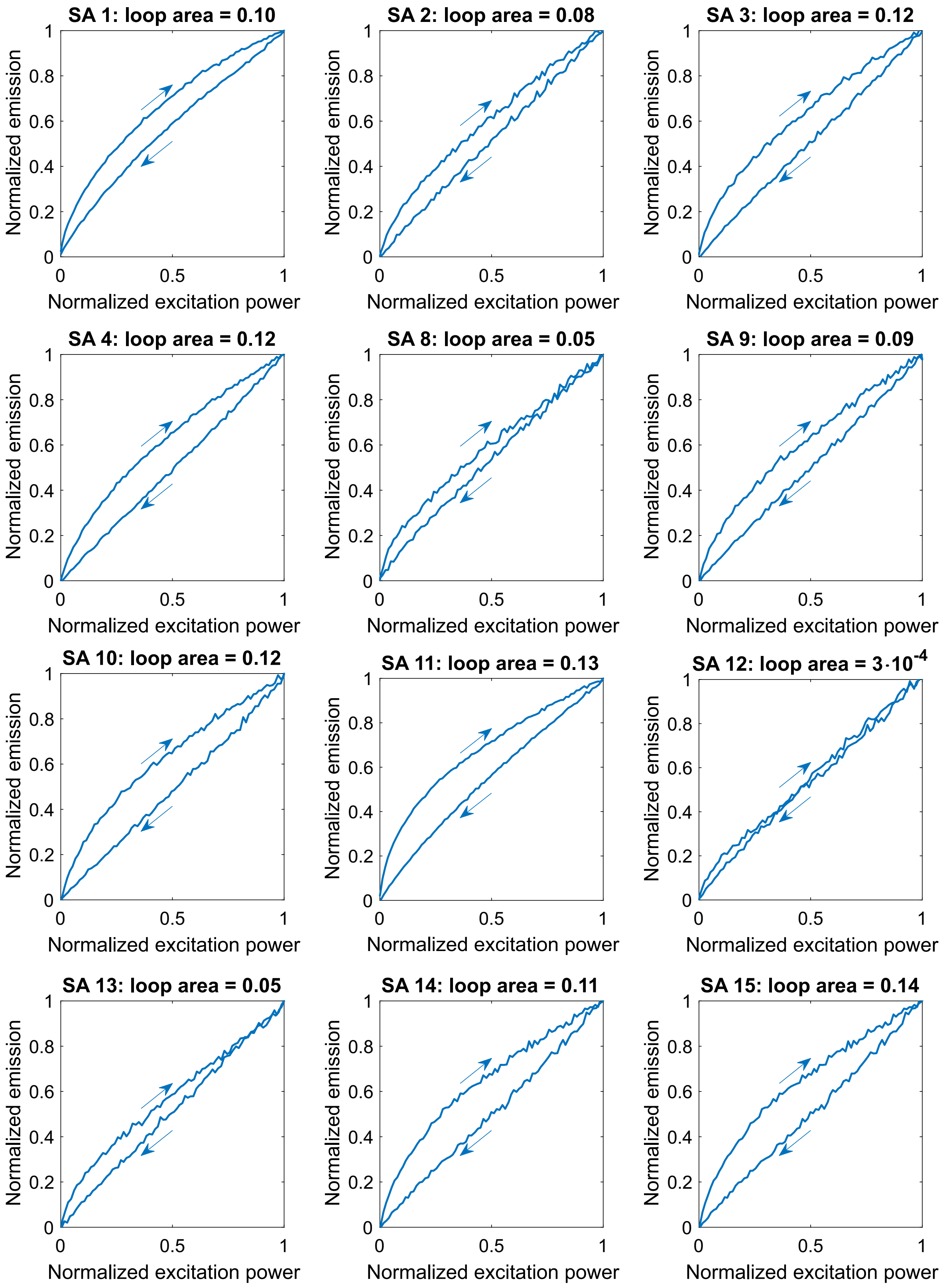

Figure S4: Hysteresis loops measured on sweeping excitation power density for SAs 1–4 and 8–15. SAs are indicated on the legends along with the areas of normalized hysteresis loops.
